# *Trichoderma harzianum* fungemia following COVID-19-related immune dysregulation in an immunocompetent patient: a case diagnosed by mNGS

**DOI:** 10.1186/s12879-026-12568-4

**Published:** 2026-01-12

**Authors:** Lin Zhou, Yanling Xu, Li Wang, Xin Li

**Affiliations:** 1 Department of Cardiovascular Medicine, The Second Hospital of Jilin University, Changchun, Jilin 130041 China; 2 Department of Respiratory Medicine, The Second Hospital of Jilin University, Changchun, Jilin 130041 China; 3 Orthopedic Medical Center, The Second Hospital of Jilin University, No. 4026 Yatai Street, Changchun, Jilin 130041 China

**Keywords:** COVID-19, Fungaemia, Immunocompetent, Metagenomic next-generation sequencing (mNGS), *Trichoderma harzianum*

## Abstract

**Background:**

*Trichoderma harzianum* is a filamentous saprophytic fungus rarely implicated in human infections. Invasive Trichoderma infections are uncommon and are typically observed in immunocompromised hosts.

**Case presentation:**

We report a 68-year-old immunocompetent male farmer who developed persistent fever and dizziness after COVID-19 infection. Initial empirical antibacterial and antiviral therapy failed to relieve symptoms. Repeated blood cultures and serological fungal tests were negative, whereas metagenomic next-generation sequencing (mNGS) of whole blood identified T. harzianum sequences (794 reads), confirming fungemia. The patient experienced severe infusion reactions to amphotericin B and visual disturbances with voriconazole, but responded well to posaconazole therapy. Fever subsided within seven days, mNGS sequence reads declined markedly, and no recurrence occurred during 12 weeks of follow-up.

**Conclusions:**

This case represents the first documented instance of T. harzianum fungemia in an immunocompetent individual following transient immune dysregulation associated with COVID-19. The report underscores the diagnostic value of mNGS in detecting rare opportunistic fungi when conventional cultures are negative and highlights posaconazole as a potential therapeutic option.

**Supplementary Information:**

The online version contains supplementary material available at 10.1186/s12879-026-12568-4.

## Introduction

Trichoderma is a saprophytic fungus widely distributed in the air, soil, trees, and even healthcare facilities. However, with the advancement of detection methods and increasing knowledge of Trichoderma infection, the number of reported cases of human infections caused by the virus has been documented. There have been reported cases of human infections caused by the virus. Genus *Trichoderma* is gradually increasing, and Trichoderma is considered an opportunistic pathogen. This assertion is particularly relevant for patients with compromised immune systems who also exhibit hematological malignancies and renal dysfunction [1, 2]. This case report presents a noteworthy instance of *Trichoderma harzianum* causing fungaemia in an immunocompetent individual, illustrating the potential for severe infections even in individuals who do not fall into traditional risk groups.

Currently, nine species of pathogens are believed to cause diseases in humans: *Trichoderma citrinoviride*, *Trichoderma harzianum*, *Trichoderma koningii*, *Trichoderma longibrachiatum*, *Trichoderma orientale*, *Trichoderma pseudoko-ningii*, *Trichoderma reesei*, and *Trichoderma viride* [3]. With improvements in diagnostic technology and improvements in our understanding of Trichoderma, the spectrum of diseases they cause has widened, including endocarditis [4, 5], pulmonary infection [6, 7], pneumonia-parapneumonic effusion [8], acute invasive sinusitis [9], and peritonitis [10].

The filamentous fungi that belong to the Genus *Trichoderma*. demonstrate characteristic tissue morphology featuring hyaline, septate filamentous structures that exhibit histopathological similarities to Aspergillus species and other etiological agents responsible for hyalohyphomycosis. This morphological convergence necessitates the implementation of culture-based isolation combined with molecular diagnostic techniques for accurate species identification. Antifungal susceptibility profiling reveals noteworthy resistance patterns, with clinical isolates showing elevated minimum inhibitory concentration (MIC) values against most conventional antifungal agents, except for echinocandins and voriconazole, in standardized in vitro assays. Epidemiological data indicate substantial associated mortality rates approaching 53% in documented infections, a concerning statistic compounded by the current absence of standardized therapeutic protocols derived from rigorous clinical trial evidence. This communication presents a clinically significant case of T. harzianum-induced fungemia, confirmed through metagenomic next-generation sequencing (mNGS) analysis, occurring in a 68-year-old immunocompetent male patient - a demographic presentation warranting particular attention given the organism’s typical association with immunocompromised hosts.

## Case presentation

A 68-year-old man with no significant medical history experienced moderate fever for 8 days, and the highest body temperature was 37.6 °C, accompanied by chills, dizziness, no night sweats, and no headache. After the oral administration of ibuprofen, his body temperature decreased to normal, and dizziness disappeared. Fever and dizziness reappeared after approximately 6 h. After receiving treatment with cephalosporin antibiotics at a private clinic for 4 days, intermittent fever persisted. Four days prior, the patient began to cough a small amount of yellow sputum. His body temperature significantly increased, reaching 39 °C. The patient went to the local hospital for a chest Computed tomography (CT) examination, which revealed left lower lung pneumonia. The patient was permitted to visit our hospital. He denied having a history of chronic illness, trauma, or travel. He is a farmer who often comes into contact with moldy plant straw and branches.

### First-stage treatment

Upon examination, his temperature was 38.5 °C, his BP was 118/86 mmHg, his heart rate was 99 beats/min, his respiration rate was 16 breaths/min, and his oxygen saturation on pulse oximetry was 98% in ambient air at rest. Moist rales were heard in the lower left lungs. The skin had not ruptured. The results of the remaining physical examinations were unremarkable.

**Fig. 1:**
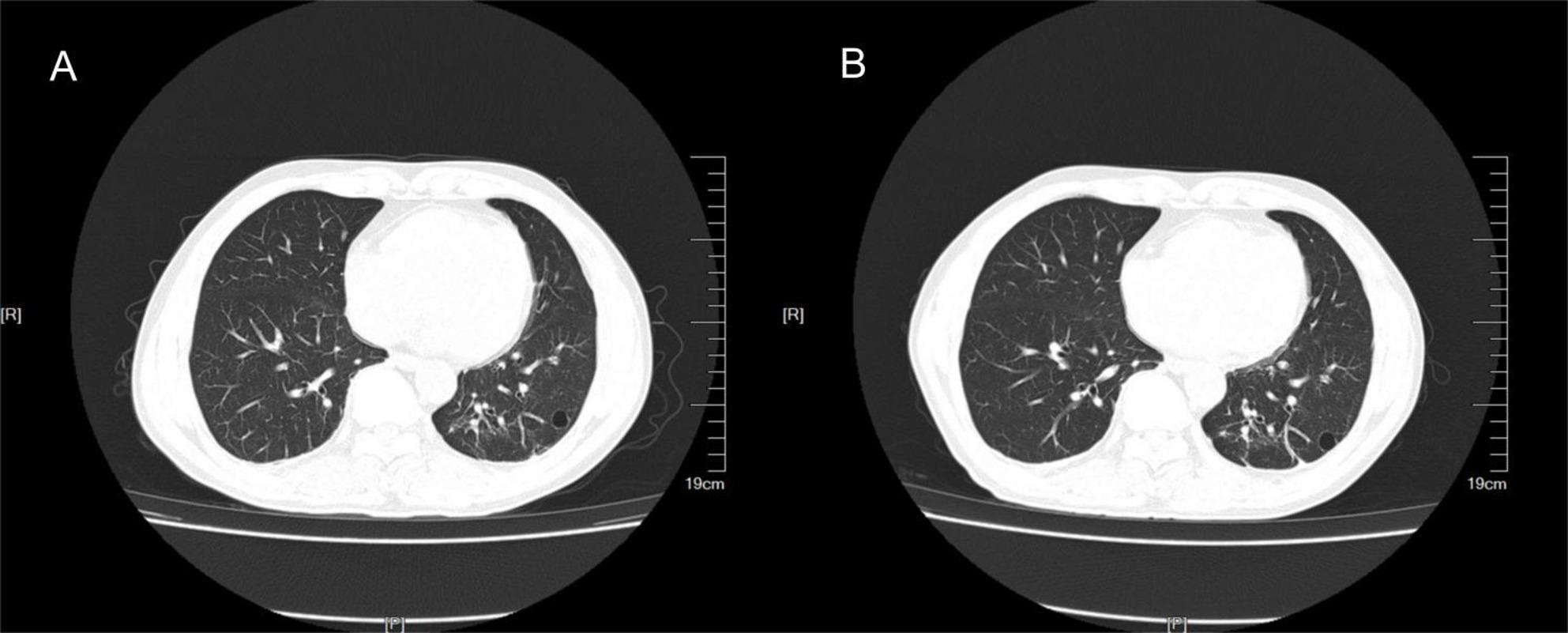
Lung CT revealed that the left inferior lobe of the lung presented a high-density shadow of a cable strip and a quasicircular transparent shadow. There was no obvious change before (**A**) or after (**B**) treatment

Arterial blood gas analysis revealed a pH of 7.46, PaCO_2_ of 40 mmHg, and PaO_2_ of 82 mmHg on room air. Laboratory examination revealed a WBC of 4.6 × 10^9^/L, with a differential of 66.3% neutrophils, 25% lymphocytes, and 7.5% monocytes (Supplementary Table 1). Hemoglobin and platelet counts were within normal ranges. Procalcitonin levels were within the normal ranges. The results for plasma (1/3)-β-D-glucan and galactomannan were negative. Tests for human immunodeficiency virus (HIV) returned negative results. CT of the chest revealed a patchy shadow in the left lower lobe (Fig. 1A). Urinary antigen tests for *Legionella pneumoniae* and *Streptococcus pneumoniae*, serological testing for *Mycoplasma pneumoniae*, and three blood cultures were negative. Pharyngeal swabs were positive for SARS-CoV-2 infection.

The patient was then diagnosed with acquired pneumonia and COVID-19. The patient received intravenous antimicrobial therapy with piperacillin and antiviral treatment with monoclavir. After 4 days, the cough and sputum were relieved, but the patient still had a fever of 37.5 °C and dizziness, which usually presented in the afternoon.

### Second-stage treatment

**Fig. 2:**
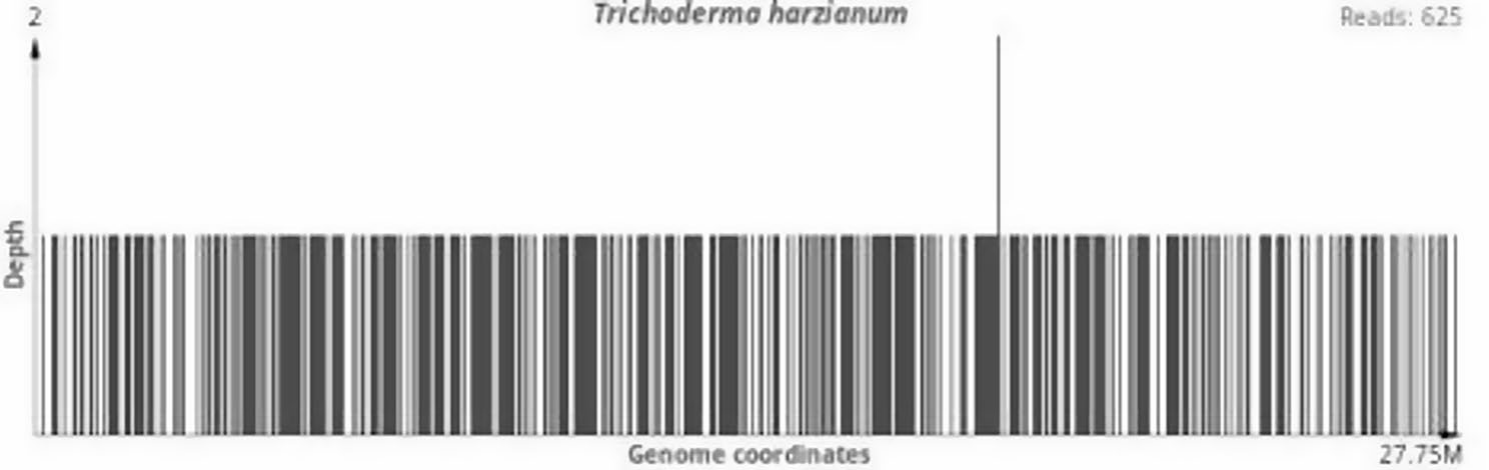
Distribution of *Trichoderma harzianum* reads across the reference genome detected by mNGS (625 reads)

**Fig. 3:**
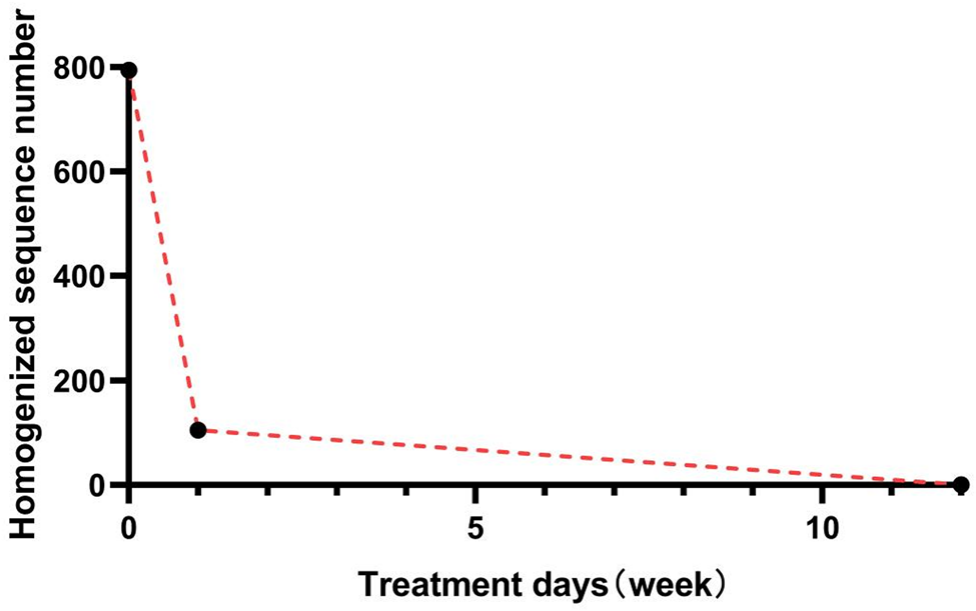
Relationship between *T. harzianum* sequence reads detected by mNGS and treatment duration in whole blood samples

As the patient still had fever, we improved upon other relevant examinations. Routine blood examination revealed a lymphocyte count of 0.8 × 10^9^/L((1.1–3.2)10 × ^9^/L), no obvious abnormality was found in the rest of the samples, and the lymphocyte classification results revealed that the CD3+, CD4+, and CD8 + T lymphocyte counts and the B and NK lymphocyte counts all decreased (Table 1). The results of the plasma procalcitonin, (1/3)-β-D-glucan, and galactomannan tests were negative, the results of the pharyngeal SARS-CoV-2 test were negative, and the results of the lung CT reexamination revealed that the shadow in the left lung was slightly absorbed (Fig. 1B). Head and abdominal CT revealed no abnormalities. Antinuclear Antibody (ANA) and Antineutrophil Cytoplasmic Antibodies (ANCA) showed no significant abnormalities. Before mNGS testing, three sets of aerobic and anaerobic blood cultures, along with urine and sputum cultures, were performed, all yielding negative results. No tissue or organ biopsy was available for histopathological examination because imaging revealed no localized lesions. therefore, whole-blood samples were tested with mNGS, which identified *T. harzianum* as the causative pathogen. Whole-blood mNGS (WillingMed Technology Co., Ltd., China) was performed, and *T. harzianum*, which was classified as a pathogenic fungus according to quality control standards, was detected (number of homogenized sequences, 794) (Fig. 2). No tissue biopsy was performed for histopathological evaluation because imaging revealed no localized lesions appropriate for the procedure sampling. The patient was a farmer and had daily exposure to moldy plant straw and branches. The patient was diagnosed with the fungus *T. harzianum*. Intravenous amphotericin B deoxycholate was started at a dose of 0.7 mg/kg/day as empirical antifungal therapy. However, after two doses, the patient experienced severe infusion-related reactions, including high fever, chills, and rigors, which required discontinuation of the drug. The treatment regimen was then switched to intravenous voriconazole, administered at 6 mg/kg twice on the first day and followed by 4 mg/kg twice daily. Unfortunately, within 48 h, the patient began to experience visual disturbances, leading to the discontinuation of voriconazole. Consequently, the antifungal therapy was changed to posaconazole, which was well tolerated. After seven days of treatment, the patient’s fever completely resolved, and his body temperature returned to normal.Repeat metagenomic next-generation sequencing (mNGS) showed a significant reduction in *T. harzianum* sequence reads (from 794 to 105), indicating successful pathogen clearance (Fig. 3). The patient completed a 12-week course of oral posaconazole without relapse or adverse effects. During three months of outpatient follow-up, no recurrence of fever or other symptoms occurred, confirming a complete recovery and stable clinical condition.

**Table 1:** Results of the immune function assay

Laboratory examination	Results
CD3+ (T lymphocytes) (52.11–81.55) %	67.88
CD3 + CD4+(T helper lymphocytes) (21.92–45.96) %	17.16↓
CD3 + CD8+(T inhibits lymphocytes) (14.19–43.41) %	41.82
CD4+/CD8+(B lymphocytes) (0.60–2.88)	0.41↓
CD3- CD19+(B lymphocytes) (5.05–20.45) %	11.27
Total lymphocyte count (1149.2-2564.7) /µL	584.0↓
CD3- CD(16 + 56)+(NK lymphocytes) (6.85–36.98) %	17.33
CD3 + T lymphocyte count (834.47-2216.80) /µL	396.42↓
CD4 + T count (395.36-1264.17) /µL	100.21↓
CD8 + T count (269.47-1059.43) /µL	244.23↓
CD3-CD19 + B count (91.53–498.00) /µL	65.82↓
CD3-CD56 + NK count (136.29-880.04) /µL	101.21↓

## Discussion

This case highlights the challenge of distinguishing bacterial from fungal infections in the context of COVID-19 coinfection. The patient initially received empirical intravenous antibacterial therapy for presumed bacterial pneumonia before pathogen identification. After mNGS confirmed *T. harzianum* fungemia, antibacterial treatment was discontinued and targeted antifungal therapy was started, leading to significant clinical improvement. To our knowledge, this is the first report of fungemia caused by T. harzianum in an immunocompetent individual. In this case, pharyngeal swab testing confirmed SARS-CoV-2 infection at admission, and the result turned negative after 10 days of antiviral treatment. However, the patient’s persistent fever and dizziness after viral clearance suggested that *T. harzianum fungemia* likely developed as a secondary infection following COVID-19 recovery. This sequence aligns with previous observations that temporary immune dysregulation after COVID-19 may make patients more vulnerable to opportunistic fungal infections.

The ability of T. harzianum to produce extracellular aspartic proteases and hydrolytic enzymes, as well as its high reproductive capacity and efficient utilization of nutrients, including amino acids, such as carbon and nitrogen sources, allows it to survive under unfavorable conditions, including growth at elevated temperatures up to 40 °C [11]. Invasive Trichoderma infections often affect the lungs, peritoneum, and central nervous system (CNS). The lungs are the primary sites of invasive Trichoderma infection in patients with malignancies [12].

As *T. harzianum* has been associated with only several human cases, one is a disseminated fungal infection that was detected in the postmortem examination of a renal transplant recipient and confirmed in culture [13], the other is *T. harzianum* peritonitis in peritoneal dialysis [14], and the other is fatal *T. harzianum* infection in a leukemic pediatric patient [11]. Our patient had no history of organ transplantation, chronic diseases, immune deficiencies, or immunosuppressants, except for being a farmer who often came into contact with moldy plant straws and branches. The patient also denied any history of skin trauma, and a physical examination did not reveal any skin damage. Therefore, clinical workers should be vigilant of fungal infections in highly immunocompromised patients and of patients who may come into contact with saprophytic fungi. Unfortunately, the general signs and symptoms of invasive fungal infections are usually atypical and nonspecific, reducing the opportunity to make an accurate clinical diagnosis [11].

However, the standard definition of invasive fungal infections requires microscopic visualization of fungal elements in tissue samples and isolation of fungi in culture [11]. The culture conditions in microbial rooms or laboratories are limited, and the culture time is extended, severely affecting treatment and prognosis. Therefore, the definition of an invasive fungal infection may rely on a combination of less specific clinical, laboratory, and radiological data [11].

While the European Organization for Research and Treatment of Cancer/Mycoses Study Group (EORTC/MSG) includes positive blood cultures as a diagnostic criterion for invasive fungal infections [12], fungal growth was not detected in blood cultures in this case. Although blood cultures demonstrate high sensitivity for microbiological pathogens, their diagnostic utility for invasive mycoses remains limited, as recent studies highlight their suboptimal sensitivity (50%) for fungal detection. Notably, negative fungal blood cultures—singular or repeated—cannot exclude disseminated fungal infections due to the inherently low sensitivity of hemocultures for fungal detection. This is exemplified by the infrequent positivity of blood cultures in cases of confirmed invasive aspergillosis or candidiasis [15], a finding further supported by a 2023 meta-analysis underscoring blood culture’s < 20% sensitivity for invasive candidiasis in immunocompromised cohorts [16].

Similarly, *Trichoderma* species infections, even when systemically disseminated, rarely yield positive blood cultures; isolated reports of such positivity predominantly involve immunocompromised patients with underlying conditions such as hematological malignancies, solid tumors, or HIV infection [12, 15–17]. Recent case series (2012–2022) have documented 4 cases of Trichoderma spp in patients receiving prolonged immunosuppressive therapy, reinforcing the association between host vulnerability and detectable fungemia [17]. Advances in molecular diagnostics, however, are shifting this paradigm. For instance, a large-scale study published in 2024, which included 546 patients, demonstrated that metagenomic next-generation sequencing (mNGS) achieved 88% sensitivity for invasive fungal infections, outperforming conventional cultures in detecting fastidious fungi [18].

As a new detection method for pathogenic microorganisms developed in recent years, mNGS has the advantages of high timeliness (doctors can obtain results within 24 h) and high sensitivity [19]. This may be a better alternative for pathogenic microbes that do not require special culture conditions, and its cost is comparable to that of laboratory detection (pathogen culture). The use of mNGS in diagnosing Trichoderma infections is supported by cases where traditional diagnostic methods were insufficient, and mNGS provided the necessary resolution to identify the pathogen accurately.

In the context of Trichoderma infections, several case reports have highlighted the challenges and outcomes associated with these infections. For instance, a case of fatal post-operative Trichoderma longibrachiatum mediastinitis and peritonitis in a pediatric patient underscores the severity of such infections in immunocompromised individuals and the need for prompt and accurate diagnosis [10]. Similarly, another report of invasive Trichoderma longibrachiatum. Infection in a neutropenic patient diagnosed with acute myeloid leukemia. emphasizes the life-threatening nature of these infections and the importance of early detection and treatment [20].

Guidelines recommend a triazole or lipid formulation of amphotericin B as the primary treatment for rare mold infections [12]. Sautour et al. reported 14 cases of human infections caused by T. *longibrachiatum*. The overall survival rate was 64%, and the site of fungal infection showed high variability. The MICs of the antifungal drugs were similar to those previously reported for amphotericin B, voriconazole, and caspofungin [7]. Another study reported the in vitro activities of nine antifungal agents against rare pathogenic fungi [21]. A limitation of this report is the absence of histopathological confirmation, since no tissue biopsy was available due to the lack of a localized lesion. However, the diagnosis of T. harzianum fungemia was strongly supported by metagenomic next-generation sequencing (mNGS) results and the patient’s rapid clinical improvement with targeted antifungal therapy.

## Conclusion

This case highlights the occurrence of Trichoderma harzianum fungemia in an immunocompetent individual following transient immune dysregulation associated with COVID-19 infection. The infection was likely facilitated by frequent environmental exposure to moldy plant materials. The case emphasizes the diagnostic challenge of rare filamentous fungal infections, especially when traditional culture methods are negative. Metagenomic next-generation sequencing (mNGS) provided rapid and accurate pathogen identification, enabling timely adjustment of antifungal therapy. The patient responded well to posaconazole, indicating that triazoles may be an effective treatment option for T. harzianum infections. More clinical evidence is needed to establish standardized diagnostic and treatment strategies for this uncommon opportunistic fungal infection.

## Electronic Supplementary Material

Below is the link to the electronic supplementary material.

Supplementary Material 1

## Data Availability

Data is provided within the manuscript or supplementary information files. For raw data， please contact Xin LI，MD，PhD，at lixin27@jlu.edu.cn.

## Abbreviations

mNGS: metagenomic next-generation sequencing
HIV: Human immunodeficiency virus
CT: Computed tomography
COVID-19: coronavirus-19
CNS: central nervous system
MIC: minimum inhibitory concentration
ANA: Antinuclear Antibody
ANCA: Antineutrophil Cytoplasmic Antibodies
