# Supplementary material for: Trichoderma harzianum fungemia following COVID-19-related immune dysregulation in an immunocompetent patient: a case diagnosed by mNGS
Source: BMC Infect Dis. 2026 Jan 12;26:298. doi: 10.1186/s12879-026-12568-4 (PMC12888569; doi:10.1186/s12879-026-12568-4)
Supplement: Supplementary file 1 — Supplementary Material 1 [file 12879_2026_12568_MOESM1_ESM.doc]

**Supplementary Table 1. Laboratory date on admission**

| Laboratory examination | Patient date |
| --- | --- |
| white blood cell count（3.5-9.5）10×9/L | 4.6 |
| Neutrophil percentage（40-75）% | 66.3 |
| Lymphocyte percentage（20-50）% | 25 |
| Lymphocyte count（1.1-3.2）10×9/L | 1.2 |
| Percentage of monocytes（3-10）% | 7.5 |
| Hemoglobin（130-175）g/L | 159 |
| platelet（125-350）10×9/L | 222 |
| Alanine aminotransferase（9-50）U/L | 10 |
| Aspartate aminotransferase（15-40）U/L | 19 |
| albumin（40-55）g/L | 45.1 |
| lactate dehydrogenase（120-250）U/L | 226 |
| Urea nitrogen（3.60-9.50）mmol/L | 6.52 |
| C-reactive protein（0-3.0）mg/L | ＜3.00 |
| IL-6（0-6.4）pg/mL | 2.51 |
| Procalcitonin＜0.5000ng/ml | 0.042 |
| Fungal (1-3) -β- d-glucan（0-100.5）pg/L | 11.6 |
| galactomannan | negative |
| Immunoglobulin-G（7.0-16.0）g/L | 12.90 |
| Immunoglobulin-M（0.4-2.3）g/L | 0.788 |
| Immunoglobulin-A（0.7-4.0）g/L | 2.41 |
| Complement-C3（0.9-1.8）g/L | 1.15 |
| Complement-C4（0.1-0.4）g/L | 0.41 |
